# Decreased IL-8 levels in CSF and serum of AD patients and negative correlation of MMSE and IL-1β

**DOI:** 10.1186/s12883-016-0707-z

**Published:** 2016-09-26

**Authors:** Raphael Hesse, Anke Wahler, Pauline Gummert, Stefanie Kirschmer, Markus Otto, Hayrettin Tumani, Jan Lewerenz, Cathrin Schnack, Christine A. F. von Arnim

**Affiliations:** Department of Neurology, University of Ulm, Helmholtzstraße 8/1, 89081 Ulm, Germany

**Keywords:** Neuroinflammation, Cytokines, Alzheimer’s disease

## Abstract

**Background:**

It is widely accepted that neuroinflammatory processes play an important role in the pathogenesis of Alzheimer’s disease (AD) and high levels of cytokines and chemokines are detected around Aβ plaques.

**Methods:**

As neuroinflammation is involved in the development and progression of AD, we measured the pro-inflammatory cytokines interleukin 1β (IL-1β), IL-8 and tumor necrosis factor α (TNF-α) in serum and cerebrospinal fluid (CSF) samples from 45 AD patients and 53 age-matched control subjects using a highly sensitive multiplex electrochemiluminescence assay. To address the association with disease progression we correlated cognitive status with cytokine levels.

**Results:**

CSF as well as serum IL-8 levels were found to be significantly lower in AD patients than in controls (*p* = 0.02). A statistically significant inverse correlation was observed between the CSF level of IL-1β and the MMSE score (r_s_ = -0.03, *p* = 0.02). We therefore stratified the AD patients by their MMSE scores into three equal groups and found that in the AD group with the most severe cognitive impairment CSF-IL-1β was significantly increased compared to age-matched controls (*p* < 0.05), whereas in the other investigated groups the increase was not statistically significant.

**Conclusion:**

Our results confirm data suggesting that cytokine alterations are involved in AD pathogenesis and may be helpful as a biomarker for monitoring disease progression.

**Electronic supplementary material:**

The online version of this article (doi:10.1186/s12883-016-0707-z) contains supplementary material, which is available to authorized users.

## Background

Alzheimer’s disease (AD) is a progressive neurodegenerative disorder and the most common form of dementia worldwide [1, 2]. The major neuropathological hallmarks of the disease are (I) extracellular senile plaques mainly composed of amyloid-β (Aβ) peptides, which are often surrounded by reactive glia and dystrophic neurites, (II) intracellular neurofibrillary tangles composed of hyperphosphorylated tau protein as well as (III) neuronal loss and synaptic dysfunction [3–5].

A growing number of studies further implicated neuroinflammation in the pathogenesis of AD [6–8]. Reactive microglia and astrocytes cluster around Aβ plaques both in the brains of individuals with AD as well as in transgenic mice [9–12] and have been suggested to promote neurodegeneration. Once activated by pathological triggers such as neuronal death or protein aggregates, microglia undergo a rapid change in morphology. They migrate to the lesion initiating an innate immune response by producing cytotoxic factors such as pro-inflammatory cytokines, chemokines and reactive oxygen species. Since neuroinflammation seems to play a role in neurodegeneration, several studies have suggested that cytokines might enhance this inflammatory process contributing to synaptic dysfunction and subsequent neuronal death. For example, interleukin (IL)-1β can be detected in reactive astrocytes surrounding Aβ deposits [13, 14] and Aβ was shown to induce the production of IL-1β [15]. Levels of IL-1β were found to be elevated in cerebrospinal fluid (CSF), plasma samples or post-mortem brain tissue of AD patients [16–19], while other studies reported no changes in IL-1β in serum or CSF of AD patients compared to controls [20, 21].

Regarding AD, the above-mentioned IL-1β as well as IL-6, IL-8, IL-10, IL-12, tumor necrosis factor (TNF)-α and transforming growth factor (TGF)-β have been intensively studied in post-mortem brain tissue, serum and CSF samples (reviewed in [22–24]). Many of these studies, however, are limited by test cohort sample size and methodological differences analyzing only post-mortem brain tissue, serum or CSF. Furthermore, the results of different studies are often inconsistent and correlations between clinical variables such as mini-mental state examination (MMSE) and cytokine levels in AD patients are often missing.

Currently, AD can be definitively diagnosed only after death by post-mortem examination of the brain [25, 26]. Neurodegeneration in AD, however, is estimated to start decades before the first clinical symptoms appear. Thus, reliable biomarkers are needed for an early diagnosis of the disease. As cytokines have already been shown to be associated with AD, the aim of this study was to further elucidate the role of cytokines in AD by simultaneous assessment of IL-1β, IL-8 and TNF-α levels in serum and CSF samples of AD patients compared with age-matched controls and to investigate whether these cytokines correlate with cognitive performance.

## Methods

### Patients

CSF and blood samples were collected at the Memory Clinic of the Department of Neurology, University Hospital Ulm, from 2003 to 2012. A total of 98 subjects were included in this study: 45 AD patients and 53 age-matched controls (Table 1). Paired CSF and serum samples were collected from 31 AD patients and 21 age-matched controls. Medical histories, as well as neurological, psychiatric, neuroradiological and neuropsychological examinations including MMSE, were obtained. AD patients were diagnosed according to the National Institute of Neurological and Communicative Diseases and Stroke–Alzheimer's Disease and Related Disorders Association criteria [27] and the *DSM-IV-TR* criteria [28]. Patients with AD showed positive CSF biomarkers (Aβ42 < 550 pg/ml, total tau > 400 pg/ml), while controls displayed a negative biomarker profile. The subgroups including the patients stratified by MMSE were equal-sized.

**Table 1:** Demographic and clinical characteristics of patients and controls included in this study

	CSF donors database		Serum donors database	
	AD (*n* = 41)	Control (*n* = 23)	AD (*n* = 36)	Control (*n* = 24)
Age, y median (IQR)	68 (66–72)	69 (63–73)	68 (66–70)	70 (64–72)
Sex, f/m (% f/m)	29/12 (70.7)	13/10 (56.5)	25/11 (69.4)	12/12 (50.0)
MMSE, median (IQR)	21.5 (17.0–24.0)*	29.0 (*n* = 12) (28.0–30.0)	21.0 (17.0–23.5)*	29.0 (*n* = 14) (28.0–30.0)
CSF Aβ42 (pg/ml), median (IQR)	472.0 (367.0–569.0)*	1014.0 (*n* = 14) (875.8–1191.8)	476.0 (373.8–577.3)*	1014.0 (*n* = 14) (875.8–1191.8)
CSF T-tau (pg/ml), median (IQR)	616.0 (371.0–1009.0)*	228.0 (*n* = 14) (204.8–279.8)	659.0 (412.8–1129.0)*	228.0 (*n* = 14) (204.8–279.8)

Data are presented as median values and interquartile ranges (IQR). 31 AD patients as well as 21 controls donated both CSF and serum. *p*-values were calculated using the Mann-Whitney test, AD vs. control *p < 0.0001

The control group of patients did not show clinical symptoms of dementia and underwent a lumbar puncture for other differential diagnostic reasons excluding acute or chronic inflammatory conditions. The final diagnoses were as follows: depression (*n* = 5), subjective cognitive impairment (*n* = 3), history of epilepsy (*n* = 2), neuropathic pain syndrome (*n* = 2); one patient each had: aneurysm, amblyacousia, myopathy, mild cognitive impairment, anxiety, cardiac insufficiency, gait abnormality, Tolosa-Hunt syndrome, stroke.

Clinical examination of the study participants did not show any signs of ongoing infection.

### Sample collection

CSF sample collection was performed using a standardized protocol as described previously [29]. Briefly, CSF was obtained by lumbar puncture into polypropylene tubes, to avoid possible adsorption of proteins to the tube wall. Samples were centrifuged at 1000 x *g* for 10 min, aliquoted and stored at -80 °C until analysis. CSF-Aβ42 and total tau protein levels were determined using commercially available INNOTEST® β-amyloid (1-42) and hTau Ag ELISA assay kits (Innogenetics, Gent, Belgium) according to the manufacturer’s instructions.

### Cytokine measurement

IL-1β, IL-8 and TNF-α were measured in CSF and serum samples using human proinflammatory cytokine assay kits and a SECTOR Imager S 6000 instrument (Mesoscale Discovery, Rockville, MA, USA) according to the manufacturer’s instructions. Samples were measured in duplicate. The assays were blind for patient identification and disease status. The detection limits were 0.28 pg/ml for IL1-β, 0.10 pg/ml for IL-8 and 0.29 pg/ml for TNF-α.

### Data analysis

The collected data failed a normality test (D'Agostino & Pearson omnibus normality test) so the comparison of groups was performed using the Mann-Whitney rank sum test (two groups) or ANOVA on ranks (> two groups). Spearman’s rank correlation analysis was used for correlation analyses. *p* < 0.05 was considered statistically significant and is indicated by an asterisk. n.s. indicates non-significant differences. The results are expressed as (median / 25^th^–75^th^ percentile).

## Results

### Decreased IL-8 but not IL-1β or TNF-α levels in CSF of AD patients

**Fig. 1:**
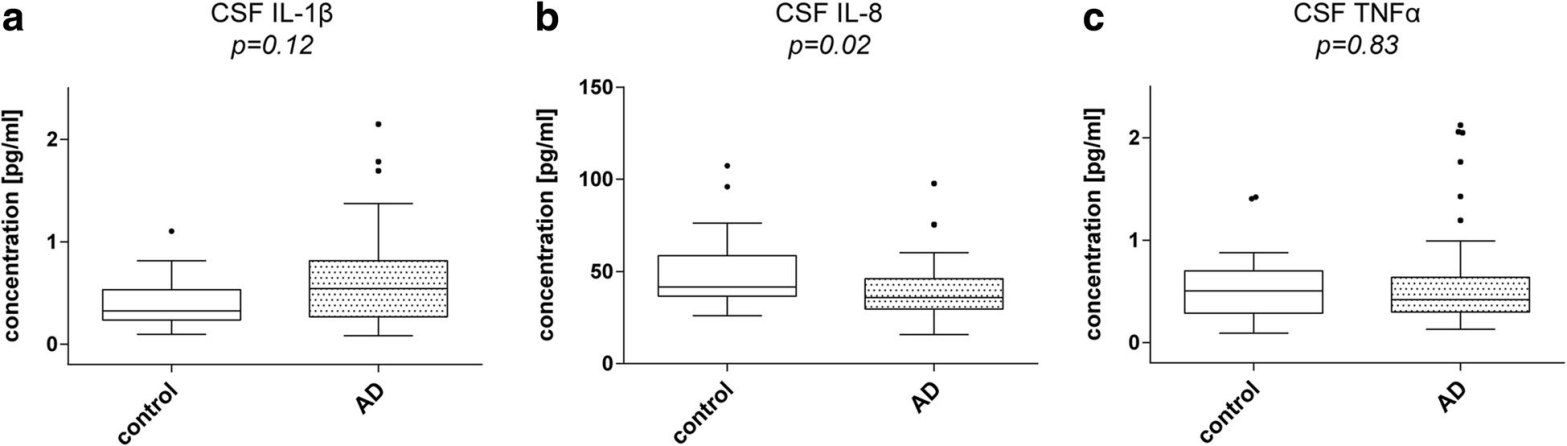
Cytokine levels in CSF samples of AD patients compared to controls. Box plots comparing CSF IL-1β, IL-8 and TNF-α levels between AD patients and age-matched controls. **a** IL-1β levels were not altered in AD patients compared to controls (*p* = 0.12). **b** IL-8 levels, by comparison, were significantly reduced in AD patients (*p* = 0.02). **c** TNF-α showed no significant difference (*p* = 0.83). Dark horizontal lines represent the mean, with the box representing the 25^th^ and 75^th^ percentiles of the observed data, the whiskers representing the 5^th^ and 95^th^ percentiles, and outliers represented by *dots*. *P* values were calculated using the Mann-Whitney Rank sum test

In our study cohort, the CSF IL-1β levels of AD patients (0.54 / 0.27–0.82 pg/ml) showed no change compared to non-demented elderly control subjects (0.33 / 0.24–0.53 pg/ml; *p* = 0.12) (Fig. 1a). IL-8 levels were found to be significantly lower in AD patients than in controls (AD: 35.0 / 29.67–46.16 pg/ml, control: 41.73 / 36.73–58.74 pg/ml; *p* = 0.02) (Fig. 1b). In the case of TNF-α, its level in CSF was not changed in AD samples (0.42 / 0.30–0.64 pg/ml) compared to control subjects (0.51 / 0.29–0.70 pg/ml; *p* = 0.83) (Fig. 1c). As depression frequently occurs already in early stages of AD and IL-8 is discussed to be involved in depressive disorders, we included subjects suffering from depression in our control cohort as well. We compared CSF IL-8 levels of control subjects suffering from a depression (39.10 / 35.71–54.84 pg/ml) with CSF IL-8 levels of non-depressed control subjects (44.01 / 35.94–67.43 pg/ml). We did not see a statistically significant difference (*p* = 0.71) (Additional file 1: Figure S1A). Further we omitted the depressed control subjects (44.01 / 35.94–67.43 pg/ml) and compared CSF IL-8 levels with these of AD patients (35.90 / 29.67–46.16 pg/ml) and observed the same difference as when the depressed control subjects were included (*p* = 0.03) (Additional file 1: Figure S1C).

### Decreased IL-8 but not IL-1β or TNF-α levels in serum of AD patients

**Fig. 2:**
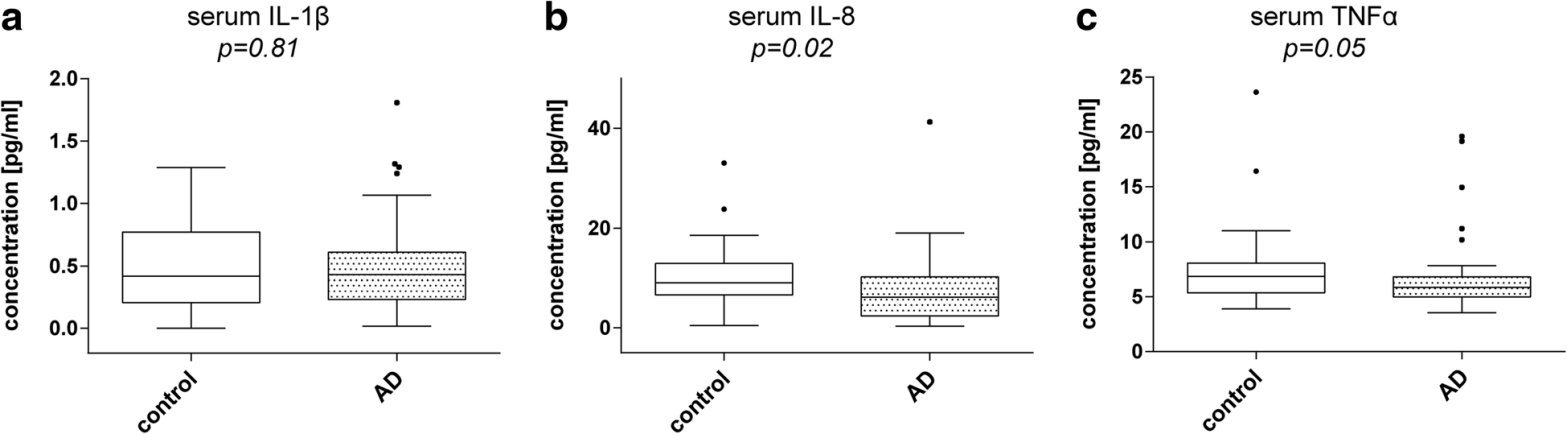
Cytokine levels in serum of AD patients and age-matched controls. Box plots comparing Serum IL-1β, IL-8 and TNF-α levels between AD patients and age-matched controls. **a** IL-1β levels were unchanged (p = 0.81). **b** IL-8 levels were significantly reduced in AD patients (*p* = 0.02). **c** TNF-α showed a slight reduction in AD patients but the observed effect was not significant (*p* = 0.05). Dark horizontal lines represent the mean, with the box representing the 25^th^ and 75^th^ percentiles of the observed data, the whiskers representing the 5^th^ and 95^th^ percentiles, and outliers represented by *dots*. *P* values were calculated using the Mann-Whitney Rank sum test

Serum IL-1β levels in AD patients (0.43 / 0.23–0.61 pg/ml) showed no differences compared to control subjects (0.42 / 0.21–0.77 pg/ml; *p* = 0.81) (Fig. 2a). IL-8 levels, by comparison, were again found to be significantly lower in AD patients than in controls (AD: 6.14 / 2.38–10.24 pg/ml, control: 9.06 / 6.62–12.94 pg/ml; *p* = 0.02) (Fig. 2b). As in CSF, TNF-α serum levels were not significantly altered in AD samples (5.87 / 5.00–6.82 pg/ml) compared to elderly controls (6.87 / 5.37–8.07 pg/ml; *p* = 0.05) (Fig. 2c). To exclude depression as confounding variable in measurement of cytokine levels in serum as well, we also compared serum IL-8 levels of control subjects suffering from depression (7.80 / 6.20–70.17 pg/ml) with serum IL-8 levels of non-depressed control subjects (9.64 / 7.00–13.13 pg/ml). We did not see a statistically significant difference (*p* = 0.31) (Additional file 1: Figure S1B). Further we again omitted the depressed control subjects (9.64 / 7.00 – 13.13 pg/ml) and compared serum IL-8 levels with these of AD patients (6.14 / 2.38–10.24 pg/ml) and observed the same difference as when the depressed control subjects were included (*p* = 0.02) (Additional file 1: Figure S1D). We concluded that in our cohort CSF as well as serum IL-8 levels are not altered in patients suffering from a depression and decided to leave them included in our cohort.

### Correlations among CSF/serum markers and MMSE score and neurodegeneration biomarkers

**Fig. 3:**
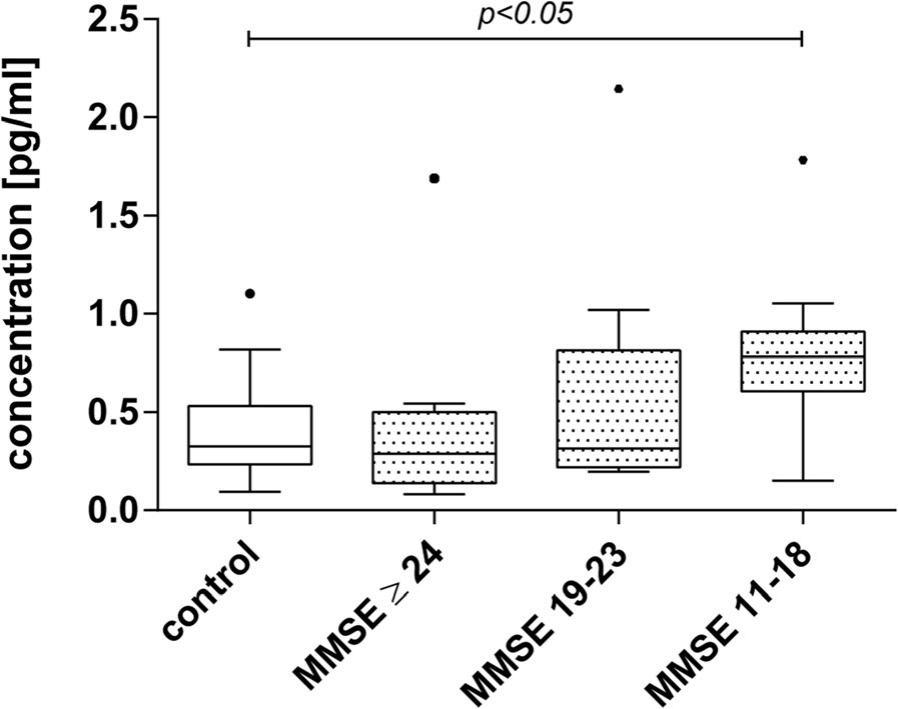
Analysis of IL-1β levels in CSF of stratified patients by MMSE. CSF IL-1β levels inversely correlated with MMSE score. To further analyze this correlation, AD patients were grouped according to their MMSE score and IL-1β levels were compared to age-matched controls. Box plots represent median levels of CSF IL-1β with the box representing the 25^th^ and 75^th^ percentiles of the observed data, the whiskers showing the 5^th^ and 95^th^ percentiles and *dots* representing outliers. The MMSE (11-18) group IL-1β was significantly increased compared to the other investigated groups (*p* < 0.05, ANOVA on Ranks, Dunn’s method)

We examined the correlations between cytokine CSF and serum levels in AD patients and their MMSE scores as well CSF Aβ42 as and CSF total tau levels as neurodegenerative markers. Only patients with complete biomarker profiles, including CSF Aβ42, CSF total tau and MMSE scores were included in correlation analysis. CSF total tau and CSF Aβ42 showed no statistically significant correlation with CSF IL-1β, CSF IL-8 or CSF TNF-α levels (Table 2). However, we observed a trend towards a correlation between CSF total tau and CSF IL-8 levels (r_s_ = 0.02, *p* = 0.06). A statistically significant inverse correlation was observed between the CSF IL-1β concentration and the MMSE score (r_s_ = -0.03, *p* = 0.02). We therefore stratified the AD patient samples by MMSE score into three equal-sized subgroups reflecting very mild, mild and moderate clinical AD stages and found that in the MMSE (11–18) group (moderate AD) IL-1β was significantly increased compared to age-matched controls (*p* < 0.05) (Fig. 3), while the mild MMSE (≥24) and the mild to moderate MMSE (18–23) showed no statistically significant difference.

**Table 2:** Correlations among CSF cytokine concentrations and MMSE, Aβ and total tau protein

	CSF IL-1β		CSF IL-8		CSF TNF-α	
	r_s_	*p*	r_s_	*p*	r_s_	*p*
MMSE	−0.33	**0.02**	0.02	0.89	−0.03	0.86
Aβ42	−0.13	0.38	0.10	0.47	−0.03	0.86
Tau	0.22	0.12	0.26	0.06	−0.08	0.56

Correlation coefficient (r_s_) and *p*-values were calculated using Spearman’s rank correlation analysis. Statistically significant results are depicted in bold

There was no association between the CSF and serum levels of the respective cytokines and also no association between the analyzed cytokines. In addition, measurements of albumin in the CSF and serum of AD and non-dementia patients excluded the possibility that dysfunction of the blood/CSF barrier influenced the level of cytokines in the CSF in the current cohort of AD patients (data not shown).

To test the diagnostic power of cytokines for the discrimination of AD patients and controls, ROC analysis was performed. Cut-offs were calculated using Youden’s index and likelihood ratio (Additional file 2: Table S1). CSF IL-8 (AUC: 0.68, 95 % CI: 0.54–0.81, *p* = 0.02) as well as serum IL-8 (AUC: 0.68, 95 % CI: 0.54–0.82, *p* = 0.02) and CSF IL1-β (AUC: 0.62, 95 % CI: 0.48–0.75, *p* = 0.12) showed limited power and therefore do not show biomarker potential as single markers. The highest discrimination power showed the ratio of CSF IL-1β and CSF IL-8 (AUC: 0.71, 95 % CI: 0.58-0.83, *p* = 0.01) (Additional file 3: Figure S2).

## Discussion

Neuroinflammation is a common feature underlying the development and progression of neurodegenerative disorders including AD (reviewed in [30–32]). Microglia, the resident innate immune cells within the central nervous system, as well as astrocytes seem to play a central role in promoting this process. When compared to controls, brains from AD patients show increased numbers of activated microglia clustering both in and around Aβ plaques [4, 10]. Microglial activation results in the production of pro-inflammatory cytokines such as IL-1β or TNF-α, which contribute to the inflammatory reaction. Several studies have analyzed the secretion of these cytokines in the serum and CSF of AD patients (for review see [22–24]). However, different study designs and types of samples lead to conflicting results that make the use of cytokines as biomarkers for AD impossible. In this study, we analyzed IL-1β, IL-8 and TNF-α in CSF and serum samples of AD patients using a highly sensitive multiplex electrochemiluminescence assay and compared the obtained concentrations with age-matched controls.

The pro-inflammatory cytokine IL-1β is believed to drive the neuroinflammatory process and has been demonstrated to be upregulated in AD and other neurodegenerative disorders [14, 18, 19]. However, there are also studies reporting that both CSF and serum IL-1β levels in AD patients are not altered [33]. In our study cohort we did not see a significant difference in IL-1β levels in CSF or serum in AD patients compared to age-matched control subjects. Interestingly however, correlation analysis revealed that CSF IL-1β levels inversely correlated with MMSE scores. We therefore split the AD group into MMSE-tertiles and identified that with increasing cognitive impairment IL-1β levels are significantly increased compared with age-matched controls. The lowest MMSE (11-18) group showed a 1.9 fold increase in CSF IL-1β, compared with controls, while the other tested groups were nearly unchanged. These results demonstrate that IL-1β might be useful as a marker for the severity of the disease. Further investigations in large cohorts are necessary to confirm our findings.

IL-8, a microglia-derived chemokine inducing chemotaxis of cells to sites of injury [34], has also been implicated in the pathogenesis of AD [35–37]. There are several studies reporting an upregulation of IL-8 in AD patients [35, 38, 39], but reductions are demonstrated as well [40–42]. In addition, a meta-analysis did not see an association suggesting the involvement of IL-8 in AD [23]. In our study cohort there was a significant reduction in CSF as well as serum IL-8 (CSF 0.84 fold, serum 0.68 fold). These conflicting results might be, at least in part, explained by differences in the examined study populations. A study from 2003 performed by Galimberti and colleagues does not provide characteristics of their study cohort [35], while Alsadany et al. include a high proportion of severe AD patients (50 % of the study cohort had MMSE score <10) [38]. In the study cohort of Zhang et al., the AD patients displayed very high total Tau levels (1425.0 ± 104.3 pg/ml) [39]. These differences clearly demonstrate the importance of standardized inclusion criteria. Another study of Galimberti et al. from 2006 showed increased CSF IL-8 levels in MCI as well as in AD patients compared to non-demented controls, whereas these levels decrease in AD patients compared to MCI patients. They further observed a trend towards lower CSF IL-8 levels in patients with MMSE scores <15 compared to patients with MMSE scores ≥15 [43]. They argue that pro-inflammatory events and intrathecal inflammation are more an initiation factor and not a consequence of AD. The absolute CSF IL-8 concentrations in our AD cohort and in the AD cohort in their study were approximately the same. The observed differences in CSF IL-8 concentrations could therefore be explained by differences in control group composition although patients with chronic or acute inflammatory conditions were excluded in both studies. Supportive evidence for our findings comes from an in-vitro study testing the effects of different chemokines on hippocampal neuronal cultures. They could show that IL-8 treatment promotes increased survival of neuronal cultures, indicating trophic effects of this chemokine [44]. Decreased IL-8 levels in AD patients could therefore be associated with declined reparative mechanism in the CNS. Interestingly, IL-8 has been shown to be involved in angiogenesis [45], and upregulation of angiogenesis in AD is hypothesized to promote neurodegeneration [46]. Whether IL-8 is downregulated as a compensatory response to upregulated angiogenesis in AD requires further examination.

TNF-α is another pro-inflammatory cytokine that is frequently reported to be regulated in AD (in any direction) [33]. Brain-derived TNF-α is mostly produced by microglia, astrocytes and neurons in response to pathological stimuli. Secreted TNF-α in turn activates TNF-α producing cells in an autocrine manner, leading to further cytokine production and astrogliosis [31]. Our measurement of TNF-α in both CSF and serum samples of AD patients and control subjects showed no significant differences between the two groups. Interestingly, studies reporting an upregulation of TNF-α often analyzed patients with severe AD, suggesting that the levels of this cytokine increase gradually but continuously during disease progression [33]. TNF-α is an unstable cytokine. For this reason, some studies have evaluated TNF-α soluble receptors (sTNF-R1 and sTNF-R2), as indirect markers of TNF-α release [47]. Therefore determination of TNF-α receptor levels could be helpful in understanding the involvement of TNF-α regulation in AD progression.

In our study cohort there was no correlation between the paired CSF and serum cytokine concentrations, indicating that the inflammatory environment in the CSF is at least in part independent from systemic cytokine production. This assumption is supported by the fact that IL-8 levels in CSF are higher than in serum, suggesting that IL-8 is more likely produced within the brain than distributed via the blood stream.

Another important point in measurement of cytokine levels is the cohort composition with regard to patient’s co-morbidities. Co-morbidities like cancer or diabetes are known to modulate inflammatory processes [48, 49]. Therefore we systematically excluded in our cohort patients suffering from these diseases. As IL-8 is discussed to be associated with depressive disorders [50], we analyzed if control subjects in our cohort suffering from depression confound CSF and serum IL-8 determination. Therefore we compared CSF and serum IL-8 levels of depressed control subjects with CSF and serum IL-8 levels of non-depressed control subjects. IL-8 levels of the compared groups did not differ significantly. Further we omitted the depressed control subjects and compared CSF and serum IL-8 levels with these of AD patients and observed approximately the same difference as when the depressed control subjects were included. Therefore we decided to leave the depressed control subjects included in our control cohort, as the CSF as well as serum IL-8 levels were not altered in patients suffering from a depression. Since it has been shown that ApoE as risk factor for AD suppress IL-1β and TNF-α secretion in an isoform-specific manner, one has to be aware the ApoE allel status when analyzing cytokines in AD patients [51]. Unfortunately we did not determine the ApoE status of all patients. Therefore we could not take this parameter into account.

The clinical significance of these cytokine measurements remains a subject of debate as the performed reported studies display great discrepancies between them making the use of inflammatory proteins as biological markers for AD unfeasible. Different inclusion criteria, analyzed sample sizes and the sensitivity of the assays used all contribute to the reported variability. Several studies have analyzed cytokine concentrations in the body fluid of AD patients with commercially available ELISA kits. In some cases, however, the cytokine levels were below the detection limit of the respective ELISA assay leading to the exclusion of a large proportion of the participants [17, 52, 53]. Furthermore, the assays used, including ELISA and multiplex kits, are quite heterogeneous leading to interassay variances. The reliability of the measured cytokine concentrations could further depend on sample handling and storage conditions [54–56]. Skogstrand et al. have demonstrated that the measurable concentrations of inflammatory markers increased in serum and plasma on storage before analysis and the longer the time of storage before centrifugation the greater the differences between serum and plasma [57]. It is therefore of importance to provide guidelines for sample collection and storage as well as for the used assays to be used under standardized conditions thus making the observed data more reliable.

As there was only a marginal correlation between CSF cytokine and neurodegeneration markers, this might reflect that IL-8 and IL-1β are independent biomarkers possibly indicating separate pathogenic mechanisms involved in AD.

## Conclusion

In conclusion, our results show significantly reduced CSF and serum IL-8 levels in AD patients as well as an inverse correlation between IL-1β concentration and MMSE score. To determine cytokine serum and CSF levels, we used a highly sensitive multiplex electrochemiluminescence assay. However, it should be kept in mind that different studies have led to different results, which might be due to several reasons including patient selection and assay methods. Because of this heterogeneity, more studies with validated assays in well-defined cohorts are needed to identify inflammatory biomarkers of AD, which could improve the accuracy of diagnosis in diagnostic panels and might lead to the development of therapeutic strategies for immune modulation.

## Additional files

Additional file 1: Figure S1. IL-8 levels in CSF and serum of depression patients compared to non-depressed subjects in control group. Box plots comparing IL-8 levels between depressed control subjects and non-depressed controls. **(A)** CSF IL-8 levels were not altered in depressed control subjects compared to non-depressed controls (*p* = 0.71). **(B)** Serum IL-8 levels were unchanged in control subjects suffering from depression compared to non-depressed controls (*p* = 0.31). **(C)** CSF IL-8 levels were significantly reduced in AD patients compared to controls, when depressed control subjects were excluded. (*p* = 0.03). **(D)** Serum IL-8 levels were significantly decreased in AD patients compared to controls, when depressed control subjects were excluded. (*p* = 0.02). Dark horizontal lines represent the mean, with the box representing the 25^th^ and 75^th^ percentiles of the observed data, the whiskers representing the 5^th^ and 95^th^ percentiles, and outliers represented by dots. P values were calculated using the Mann-Whitney Rank sum test. (TIF 15013 kb) Additional file 2: Table S1. Characteristics of chemokines for AD in ROC analysis. Sensitivity is defined as the fraction of those with the disease correctly identified as positive by the test. Specificity is defined as the fraction of those without the disease correctly identified as negative by the test. Youden’s index was calculated as follows: sensitivity + specificity – 1. Likelihood ratio equals sensitivity/(1 – specificity). AUC: area under the curve, CI: 95 % confidence interval. (DOCX 16 kb) Additional file 3: Figure S2. ROC curve analysis. X-axis: specificity (false positive rate), Y-axis: sensitivity (true positive rate), **(A)** CSF IL-8, AUC: 0.68, CI: 0.54-0.81, *p* = 0.02 **(B)** serum IL-8, AUC: 0.68, CI: 0.54-0.82, *p* = 0.02 **(C)** CSF IL-1β, AUC: 0.62, CI: 0.48-0.76, *p* = 0.12 **(D)** ratio CSF IL-1β/CSF IL-8, AUC: 0.71, CI: 0.58-0.83, *p* = 0.01. AUC: area under the curve, CI: 95 % confidence interval. Further characteristics of ROC analysis are depicted in Additional file 2: Table S1. (TIF 16619 kb)

## Abbreviations

AD: Alzheimer’s disease
Aβ: Amyloid β
CSF: Cerebrospinal fluid
IL: Interleukin
MMSE: Mini-mental state examination
TNF-α: Tumor necrosis factor α
