# Supplementary figures and images for: Decreased IL-8 levels in CSF and serum of AD patients and negative correlation of MMSE and IL-1β
Source: BMC Neurol. 2016 Sep 26;16:185. doi: 10.1186/s12883-016-0707-z (PMC5037590; doi:10.1186/s12883-016-0707-z)

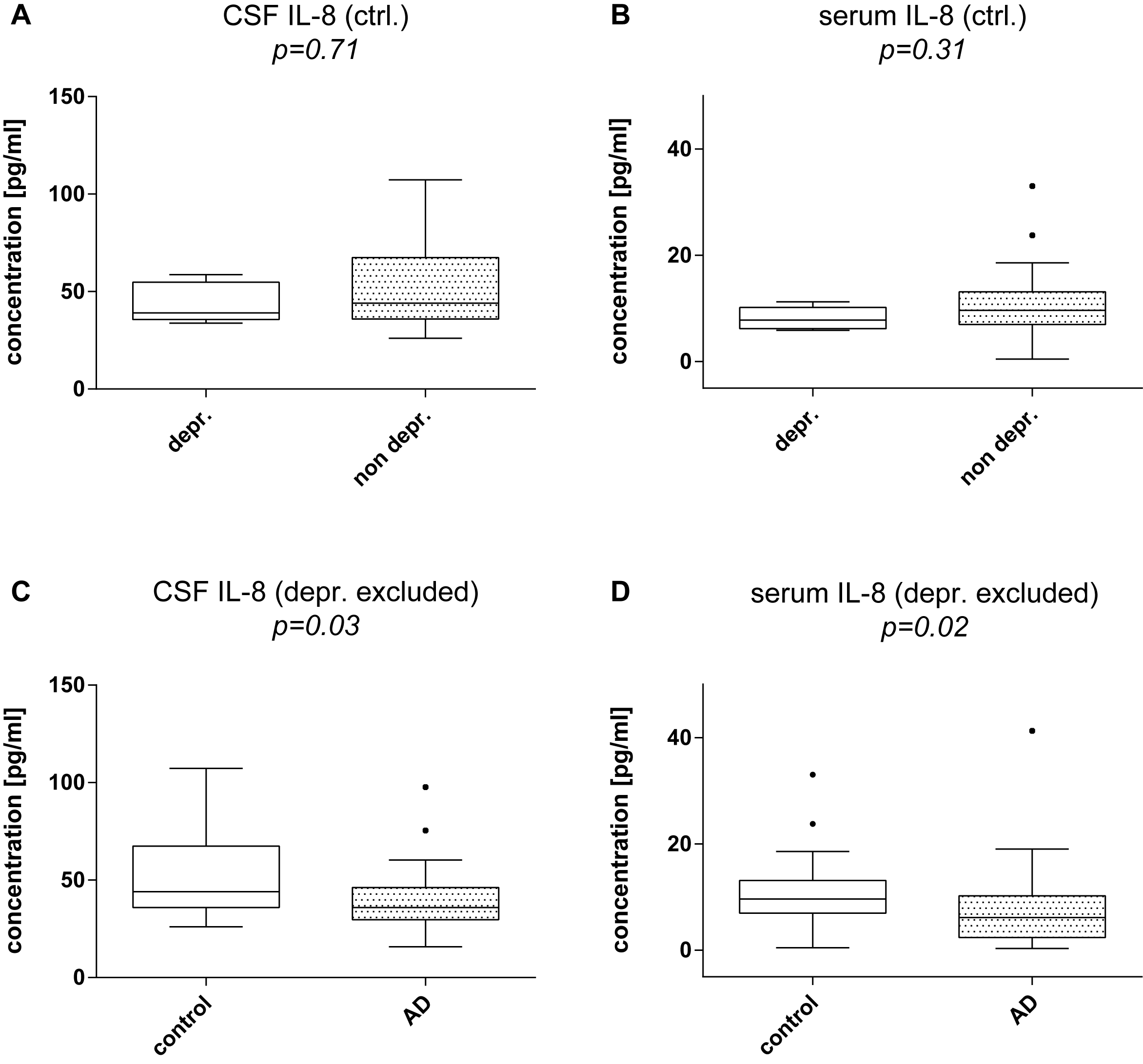

Supplement: Additional file 1: Figure S1. — IL-8 levels in CSF and serum of depression patients compared to non-depressed subjects in control group. Box plots comparing IL-8 levels between depressed control subjects and non-depressed controls. (A) CSF IL-8 levels were not altered in depressed control subjects compared to non-depressed controls (p = 0.71). (B) Serum IL-8 levels were unchanged in control subjects suffering from depression compared to non-depressed controls (p = 0.31). (C) CSF IL-8 levels were significantly reduced in AD patients compared to controls, when depressed control subjects were excluded. (p = 0.03). (D) Serum IL-8 levels were significantly decreased in AD patients compared to controls, when depressed control subjects were excluded. (p = 0.02). Dark horizontal lines represent the mean, with the box representing the 25th and 75th percentiles of the observed data, the whiskers representing the 5th and 95th percentiles, and outliers represented by dots. P values were calculated using the Mann-Whitney Rank sum test. (TIF 15013 kb) [file 12883_2016_707_MOESM1_ESM.tif]

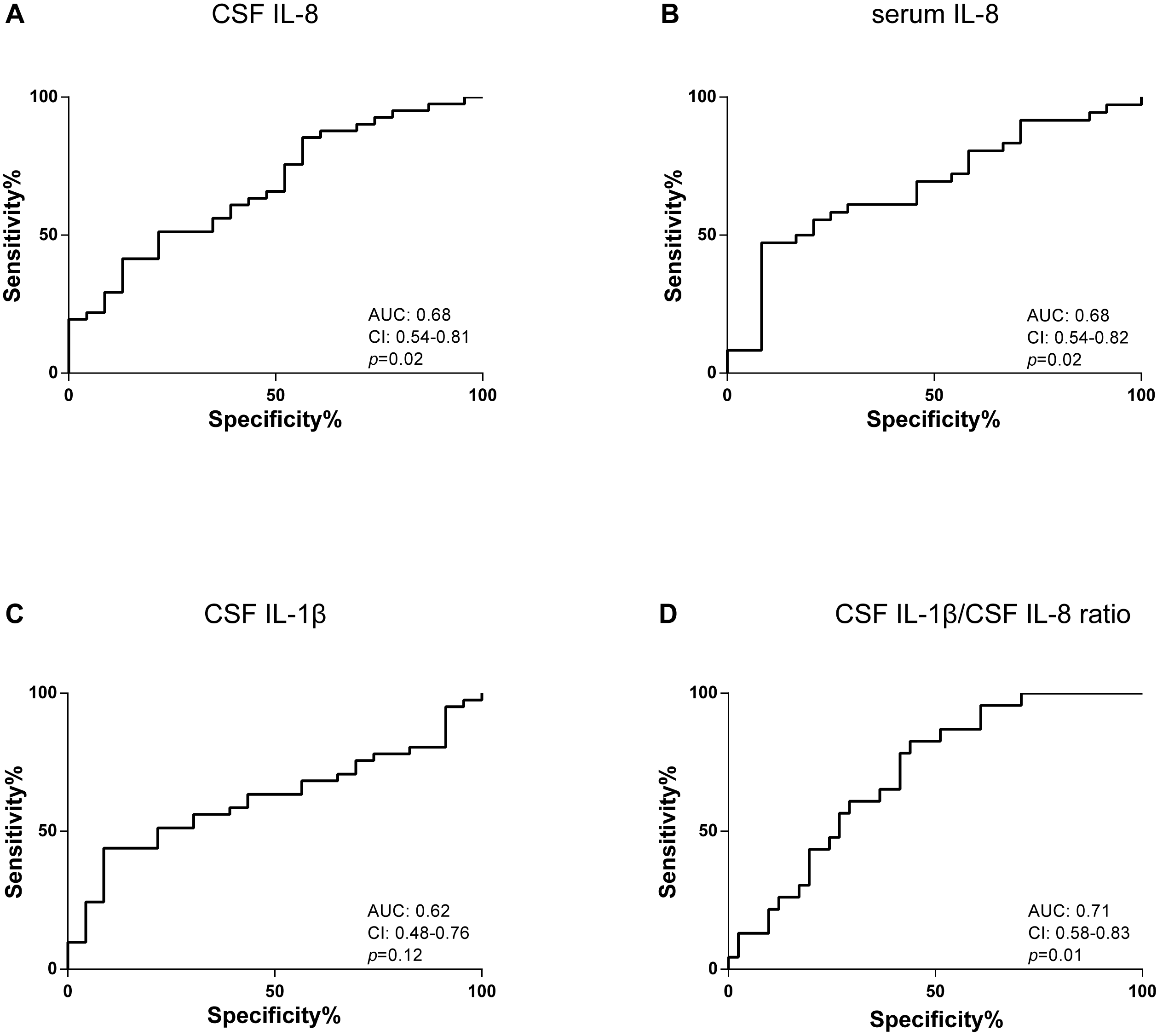

Supplement: Additional file 3: Figure S2. — ROC curve analysis. X-axis: specificity (false positive rate), Y-axis: sensitivity (true positive rate), (A) CSF IL-8, AUC: 0.68, CI: 0.54-0.81, p = 0.02 (B) serum IL-8, AUC: 0.68, CI: 0.54-0.82, p = 0.02 (C) CSF IL-1β, AUC: 0.62, CI: 0.48-0.76, p = 0.12 (D) ratio CSF IL-1β/CSF IL-8, AUC: 0.71, CI: 0.58-0.83, p = 0.01. AUC: area under the curve, CI: 95 % confidence interval. Further characteristics of ROC analysis are depicted in Additional file 2: Table S1. (TIF 16619 kb) [file 12883_2016_707_MOESM3_ESM.tif]
