# Supplementary material for: Decreased IL-8 levels in CSF and serum of AD patients and negative correlation of MMSE and IL-1β
Source: BMC Neurol. 2016 Sep 26;16:185. doi: 10.1186/s12883-016-0707-z (PMC5037590; doi:10.1186/s12883-016-0707-z)
Supplement: Additional file 2: Table S1. — Characteristics of chemokines for AD in ROC analysis. Sensitivity is defined as the fraction of those with the disease correctly identified as positive by the test. Specificity is defined as the fraction of those without the disease correctly identified as negative by the test. Youden’s index was calculated as follows: sensitivity + specificity – 1. Likelihood ratio equals sensitivity/(1 – specificity). AUC: area under the curve, CI: 95 % confidence interval. (DOCX 16 kb) [file 12883_2016_707_MOESM2_ESM.docx]

| **Additional table 1:** Characteristics of chemokines for AD in ROC analysis | | | | |
| --- | --- | --- | --- | --- |
|  | **IL-8 CSF** | **IL-8 serum** | **IL-1β CSF** | **IL-1β/IL-8 CSF ratio** |
| **AUC** | 0.68 | 0.68 | 0.62 | 0.71 |
| **95% CI** | 0.54-0.81 | 0.54-0.82 | 0.48-0.76 | 0.58-0.83 |
| ***p*-value** | 0.02 | 0.02 | 0.12 | 0.01 |
| **Youden’s index** | 0.29 | 0.39 | 0.35 | 0.39 |
| **Cut-off** | <36.31 pg/ml | <5.74 pg/ml | >0.71 pg/ml | <0.003 |
| **Sensitivity (%)** | 51.22 | 47.22 | 43.9 | 82.61 |
| **95% CI** | 35.13-67.12 | 30.41-64.51 | 28.47-60.25 | 61.22-95.05 |
| **Specificity (%)** | 78.26 | 91.67 | 91.3 | 56.1 |
| **95% CI** | 56.30-92.54 | 73.00-98.97 | 71.96-98.93 | 39.75-71.53 |
| **Likelihood ratio** | 5.05 | 5.67 | 5.61 | 5.35 |
| **Cut-off** | <26.71 pg/ml | <5.74 pg/ml | >0.82 pg/ml | <0.01 |
| **Sensitivity (%)** | 21.95 | 47.22 | 24.93 | 13.04 |
| **95% CI** | 10.56-37.61 | 30.41-64.51 | 12.63-40.30 | 2.78-33.59 |
| **Specificity (%)** | 95.65 | 91.67 | 95.65 | 97.56 |
| **95% CI** | 78.05-99.89 | 73.00-98.97 | 78.05-99.89 | 87.14-99.94 |
| AUC: area under the curve | | | | |
